# Supplementary material for: Content-rich biological network constructed by mining PubMed abstracts
Source: BMC Bioinformatics. 2004 Oct 8;5:147. doi: 10.1186/1471-2105-5-147 (PMC528731; doi:10.1186/1471-2105-5-147)
Supplement: Additional File 5 — The original Chilibot query results of the term "long-term potentiation (LTP)" and 22 other terms, limiting the latest references analyzed to the years 1990, 1995, 2000, and 2004. [file 1471-2105-5-147-S5.bz2 › chilibotAdditionalFile5/ltp2000/html/PKA_ACTININ.html]

 


 **PKA** and **ACTININ** 
  
Found 2 abstracts in PubMed,  **2 abstracts were retrieved and analyzed**.  


---

 Search Google  |
 PDF files only 
|  EDU domain only 

---

- J Cell Biol, 1998   **A kinase anchoring protein 100 AKAP100 is localized in multiple subcellular compartments in the adult rat heart.** .
  Stimulation of beta adrenergic receptors activates type I and II cyclic AMP dependent protein kinase A, resulting in phosphorylation of various proteins in the heart.
  It has been proposed that **PKA** II compartmentalization by A kinase anchoring proteins AKAPs regulates cyclic AMP dependent signaling in the cell.
  We investigated the expression and localization of AKAP100 in adult hearts.
  By immunoblotting, we identified AKAP100 in adult rat and human hearts, and showed that type I and II regulatory RI and II subunits of **PKA** are present in the rat heart.
  By immunofluorescence and confocal microscopy of rat cardiac myocytes and cryostat sections of rat left ventricle papillary muscles, we localized AKAP100 to the nucleus, sarcolemma, intercalated disc, and at the level of the Z line.
  After double immunostaining of transverse cross sections of the papillary muscles with AKAP100 plus alpha **actinin** specific antibodies or AKAP100 plus ryanodine receptor specific antibodies, confocal images showed AKAP100 localization at the region of the transverse tubule junctional sarcoplasmic reticulum.
  RI is distributed differently from RII in the myocytes.
  RII, but not RI, was colocalized with AKAP100 in the rat heart.
  Our studies suggest that AKAP100 tethers **PKA** II to multiple subcellular compartments for phosphorylation of different pools of substrate proteins in the heart.

  - Cell Motil Cytoskeleton, 1994   **Regulation of motility and cytoskeletal organization of rat bladder carcinoma cells by cyclic AMP.** .
    Cyclic AMP cAMP has been implicated in the regulation of movement of certain cultured cell types.
    We have studied the effects of cAMP on epithelial cell motility using serum free NBT II cells, derived from a rat bladder carcinoma.
    The random movement of these cells on type I collagen was reduced upon elevation of intracellular cAMP by several means and this effect was reversible.
    Alterations in the organization of the cytoskeletal proteins F actin and alpha **actinin** occurred concurrently with the reduction in motility, and the arrangement of these proteins resembled that seen in non motile cells on glass.
    In addition, pretreatment of cells with KT5720, a cAMP dependent protein kinase **PKA** specific inhibitor, prevented the dibutyryl cAMP induced reduction in cell movement as well as the associated cytoskeletal changes.
    These results suggest that elevation of **PKA** is responsible for the observed effects on cell motility and cytoskeletal reorganization and demonstrate a role for **PKA** in the regulation of cell motility in this system.
